# Erratum: Dietary Intervention in Pregnant Women with Gestational Diabetes; Protocol for the DiGest Randomised Controlled Trial; *Nutrients* 2020, *12*, 1165

**DOI:** 10.3390/nu12061793

**Published:** 2020-06-17

**Authors:** Laura C Kusinski, Helen R Murphy, Emanuella De Lucia Rolfe, Kirsten L. Rennie, Linda M. Oude Griep, Deborah Hughes, Roy Taylor, Claire L. Meek

**Affiliations:** 1 Institute of Metabolic Science, University of Cambridge, CB2 0QQ, UK; lck34@medschl.cam.ac.uk (L.C.K.); djh251@medschl.cam.ac.uk (D.H.); 2 Cambridge Universities NHS Foundation Trust, Cambridge, CB2 0QQ, UK; helen.murphy@uea.ac.uk; 3 Norwich Medical School, University of East Anglia, Norwich, NR4 7UQ, UK; 4 NIHR Cambridge Biomedical Research Centre – Diet, Anthropometry and Physical Activity Group, MRC Epidemiology Unit, Institute of Metabolic Science, University of Cambridge, CB2 0QQ, UK; emanuella.de-lucia-rolfe@mrc-epid.cam.ac.uk (E.D.L.R.); Kirsten.Rennie@mrc-epid.cam.ac.uk (K.L.R.); Linda.OudeGriep@mrc-epid.cam.ac.uk (L.M.O.G.); 5 Institute of Cellular Medicine, University of Newcastle, NE4 5PL, UK; roy.taylor@ncl.ac.uk

The authors would like to correct an error in a recent published paper [1]. Due to the misunderstanding of the study number and ISRCTN number, the authors reported the ISRCTN number incorrectly. The ‘(ISRCTN 37866)’ has been changed to ‘(ISRCTN65152174) in Section 2.1. The ISRCTN number has also been changed in the ‘Funding’ and ‘Acknowledgements’ section of the manuscript.

The authors apologize for any inconvenience caused to the readers by the change. The change does not affect the scientific results.
